# Disruption of Axonal Transport Perturbs Bone Morphogenetic Protein (BMP) - Signaling and Contributes to Synaptic Abnormalities in Two Neurodegenerative Diseases

**DOI:** 10.1371/journal.pone.0104617

**Published:** 2014-08-15

**Authors:** Min Jung Kang, Timothy J. Hansen, Monique Mickiewicz, Tadeusz J. Kaczynski, Samantha Fye, Shermali Gunawardena

**Affiliations:** Department of Biological Sciences, The State University of New York at Buffalo, Buffalo, New York, United States of America; Institut Curie, France

## Abstract

Formation of new synapses or maintenance of existing synapses requires the delivery of synaptic components from the soma to the nerve termini via axonal transport. One pathway that is important in synapse formation, maintenance and function of the Drosophila neuromuscular junction (NMJ) is the bone morphogenetic protein (BMP)-signaling pathway. Here we show that perturbations in axonal transport directly disrupt BMP signaling, as measured by its downstream signal, phospho Mad (p-Mad). We found that components of the BMP pathway genetically interact with both kinesin-1 and dynein motor proteins. Thick vein (TKV) vesicle motility was also perturbed by reductions in kinesin-1 or dynein motors. Interestingly, dynein mutations severely disrupted p-Mad signaling while kinesin-1 mutants showed a mild reduction in p-Mad signal intensity. Similar to mutants in components of the BMP pathway, both kinesin-1 and dynein motor protein mutants also showed synaptic morphological defects. Strikingly TKV motility and p-Mad signaling were disrupted in larvae expressing two human disease proteins; expansions of glutamine repeats (polyQ77) and human amyloid precursor protein (APP) with a familial Alzheimer's disease (AD) mutation (APPswe). Consistent with axonal transport defects, larvae expressing these disease proteins showed accumulations of synaptic proteins along axons and synaptic abnormalities. Taken together our results suggest that similar to the NGF-TrkA signaling endosome, a BMP signaling endosome that directly interacts with molecular motors likely exist. Thus problems in axonal transport occurs early, perturbs BMP signaling, and likely contributes to the synaptic abnormalities observed in these two diseases.

## Introduction

Pre-synaptic components, such as precursors of synaptic vesicles, active zone compartments, mitochondria and proteins essential for synaptic vesicle release must be transported down the axon to the nerve terminals by the anterograde motor kinesin-1 [1]–[4]. Upon arrival at the nerve terminal, cargo-loaded vesicles must undergo fusion with the plasma membrane to assemble active zones and reconstitute synaptic vesicles [5]–[8]. Work has shown that bone morphogenetic protein (BMP) growth factors regulate the development, growth and function of synapses in Drosophila via retrograde signaling [9]. Interestingly, a mutation of the dynein–dynactin motor p150/glued disrupted retrograde axonal transport of activated BMP as assayed by the loss of its downstream signal phospho Mad (p-Mad) accumulation in motor neuron nuclei [10], indicating that perhaps this signal could be incorporated into a signaling endosome that is transported by dynein motors [9], similar to the signaling endosome NGF-TrkA in neurotrophin signaling [11]. However, whether such a BMP signaling endosome exists and whether this complex is transported via a direct interaction with molecular motors is unclear. Further, since the BMP ligands and receptors are expressed in multiple cells in the CNS [10]–[13] how BMP signaling at the CNS plays a role in normal NMJ development and function at the distal ends of neurons is also unknown.

In many neurodegenerative diseases problems in axonal transport and synapse function have been reported long before the onset of classical disease pathologies. However the mechanisms of how defects in axonal transport directly contribute to synaptic dysfunction is unknown. In Huntington's disease (HD) mouse models, abnormal plasticity was seen before signs of disease or neuronal loss [14]–[16]. Human studies revealed synaptic dysfunction decades before clinical diagnosis in HD carriers [17]. Post-mortem studies indicated that the first clinical symptoms appear in the absence of overt neuronal loss [17], suggesting that impaired cognition was likely caused by axonal and synaptic dysfunction rather than cell death. Further, protein aggregates in neurites were observed in both transgenic HD mice and in HD-affected human brains long before the onset of clinical problems [17]–[20]. Dystrophic striatal and corticostriatal neurites in HD exhibited blocked axons: accumulations of vesicles and organelles in swollen axonal projections and in termini [21], [22]. Huntingtin accumulations were also found in striatal projection neurons in transgenic and knockin mouse models of HD and in human HD brains [23]. However whether these axonal abnormalities and synaptic dysfunction are the result of defects in long distance axonal transport is unclear.

Synapse loss has also been observed in Alzheimer's disease (AD) together with axonal transport defects. In AD brains significant synapse loss was seen in patients with mild cognitive impairment indicating that synaptic changes are critical for AD pathogenesis [24]. In animals, Aβ oligomers inhibited LTP and facilitated LTD, induced synapse loss and cognitive impairments [25]. Evidence also suggested that cholinergic disconnection and the amyloid deposition observed in AD and in AD mouse models could be related to defects in axonal transport [26]–[31]. The identification of axonal defects in early AD and in AD models was consistent with previously reported cytoskeletal and neuritic abnormalities [32]–[34], and supports the hypothesis that impaired axonal transport and synaptic alterations play a critical role in the pathogenesis of AD [35]–[37]. However the mechanistic link between axonal transport defects and synaptic dysfunction is unclear.

Using genetic analysis in Drosophila, here we tested the hypothesis that defects in long distant transport within axons directly contribute to synaptic defects by perturbing the BMP signaling pathway, a pathway essential for synapse maintenance and function. Our observations suggest that normally BMP components are transported within axons by associating with molecular motors. While anterograde motors transport BMP ligands and receptors from the soma or cell body to the nerve terminal, retrograde motors contribute to the motility of activated BMP signals from the nerve terminal towards the cell body, and this pathway is disrupted in two neurodegenerative diseases. Thus, our findings provide new insight into the pathological propagation of disease in two neurodegenerative diseases, namely that defects in long distant transport likely is the earliest contributor to the synaptic abnormalities observed in these two human neurodegenerative diseases.

## Results

### Disruption of axonal transport by loss of function of kinesin-1 and dynein causes abnormalities in synaptic morphology

**Figure 1:**
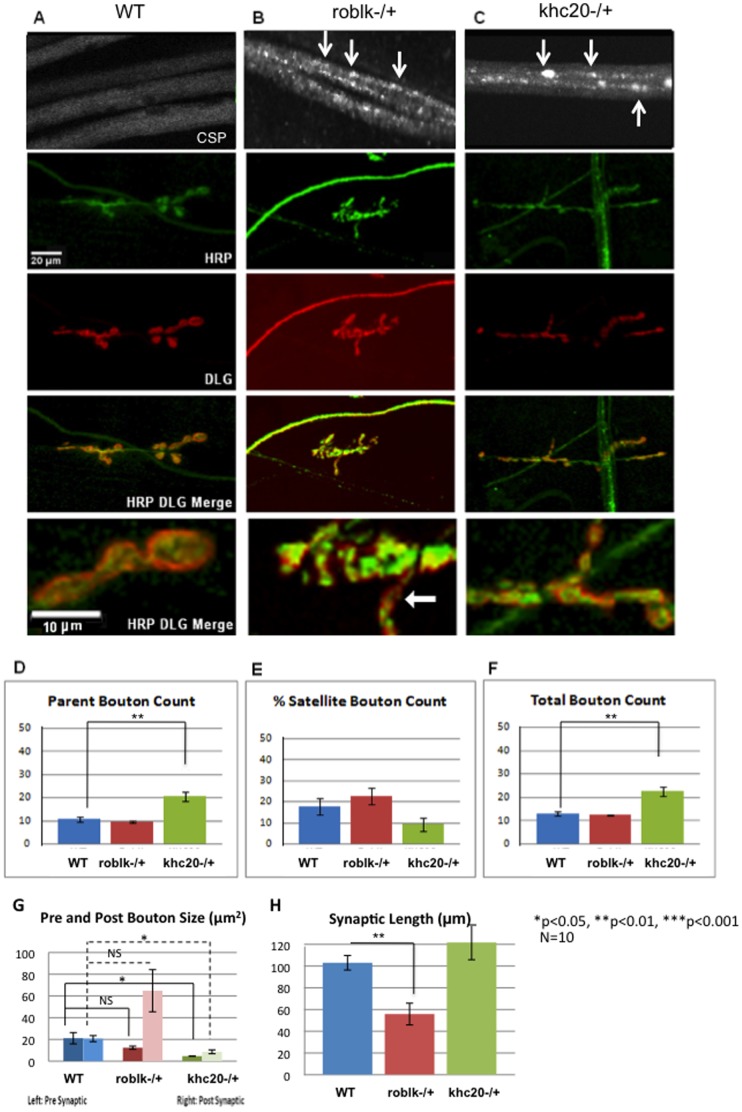
Loss of function of kinesin-1 and dynein causes abnormalities in synaptic morphology. **A:** Wild type larval segmental nerves show smooth staining with the synaptic vesicle marker cystein string protein, (CSP). Wild type NMJs from muscle 6/7 stained with the pre-synaptic marker HRP (green) and the post-synaptic marker DLC (red). Bar = 10 µm. In wild type, the post synaptic subsynaptic reticulum surrounds the pre synaptic as seen with DLG (red) in the enlarged image. Bar = 20 µm. **B:** roblk−/− larval segmental nerves show axonal accumulations with CSP (arrows). Defects in synaptic morphology are seen in NMJs from roblk−/− mutants with both pre and post synaptic markers. Note that the enlarged image shows some boutons with only the post-synpatic marker DLG (red) while the pre-synaptic marker HRP (green) was decreased (arrow). **C:** khc20−/− larval segmental nerves show axonal accumulations (arrows). Defects in synaptic morphology are seen in NMJs from khc20−/− mutants with both the pre and post synaptic markers. Note that the enlarged image shows only some boutons contain the pre-synaptic marker (green) while the post-synaptic marker appears to be decreased. **D:** Quantitative analysis of parent bouton numbers. NMJs from khc20−/− show a significant increase in the number of parent boutons (p = 0.0094) compared to wild type (WT). Y axis depicts the average number of parent boutons from 10 larvae. **E:** The percent of satellite boutons (y = axis) do not show significant changes between WT, roblk−/− (p = 0.488) or khc20−/− (p = 0.780) larvae. **F:** The total number of boutons was significantly increased in khc20−/− (p = 0.008) compared to wild type. The total number of boutons include parent and satellite boutons (y axis). **G:** Quantitative analysis of the synaptic bouton area show significant reduction in the pre-synaptic bouton areas in NMJs from khc20−/− (p = 0.0509) compared to wild type. Significant reduction is also observed in the post-synaptic bouton areas in khc20−/− NMJs (p = 0.016) compared to wild type. Although an increase is seen in the post-synaptic bouton areas in roblk−/− NMJs compared to wild type this effect is not significant (NS, p = 0.064). Areas (µm^2^) were measured using NIH image for pre and post syanpse and averaged from 10 larvae. **H:** Synaptic length quantification shows a significant decrease in the synaptic length for roblk−/− (p = 0.0102) compared to wild type. Synaptic length was measured in µm within the corresponding muscle area to avoid any bias that might arise due to a variation in muscle size among different larvae and genotypes. Y = average synaptic length. For statistical analysis ANOVA was used followed by post-hoc analysis using the Bonferroni's test. N = 10 larvae.

In Drosophila, loss of function mutants of receptors (wit, tkv, sax) and ligands (mad, med) in the BMP signaling pathway show reduced numbers of synaptic boutons with aberrant morphology, accompanied by defects in transmitter release [10], [38]–[43]. While larvae carrying mutations of kinesin-1 or dynein show axonal blockages [44]–[46], [63] it is unclear whether motor protein mutants also contain similar synaptic defects. While components of pre synapses and proteins essential for synaptic vesicle release are transported down the axon by fast axonal transport, little is known about the direct relationship between axonal transport and the integrity of synapses. To study synapse integrity in motor protein mutants, where axonal transport is severely disrupted, we examined NMJs from wandering 3^rd^ instar larvae. Specifically type 1 synaptic boutons between muscle 6 and 7 at larval abdominal segments A4–5 were examined using antibodies against pre and post synaptic markers. NMJs undergo rapid structural and functional growth in the few days of larval development. During this time the numbers of synaptic boutons increase several fold to keep up with the growing muscle [41], [47]–[49]. We observe that this coordinated growth was defective in motor protein mutants (Figure 1).

A characteristic feature of wild-type NMJs is that synaptic boutons within a branch resemble a string of beads, with boutons connected to one another by a short neuritic process [6], [50], which are named parent boutons (Figure S1, arrow heads). In certain mutants, small “satellite” boutons have been observed to appear to bud off from a central “parent” bouton of normal appearance [51] (Figure S1, arrows). In motor protein mutants these features were severely affected (Figure 1ABC). In kinesin-1 loss of function mutants (khc20−/−), the number of parent boutons was greatly increased (p<0.05) compared to wild type (Figure 1D). These increases lead to the significant increase seen in the total number of boutons in khc20−/− mutants (Figure 1F), while no change was seen in the number of satellite boutons (Figure 1E). Further, the pre synaptic bouton size assayed using the pre synaptic marker HRP was significantly reduced (p<0.05) in khc20−/− mutants compared to wild type (Figure 1G). In contrast to these observations, loss of the dynein motor (roblk−/−) did not show any dramatic change to the number of parent and satellite boutons (Figure 1D). However, the pre synaptic bouton size in roblk−/− mutant larvae appeared to be decreased but this reduction was not significant (Figure 1G, p = 0.194).

Further examination of the synaptic morphology in motor protein mutants using DLG, the marker for the post synaptic subsynaptic reticulum (SSR) revealed significant changes to the post synapses of motor protein mutants. In wild type NMJs, DLG was seen virtually in all boutons including satellite boutons, which were surrounded by SSR (Figure 1A) [52]. In contrast, the post synaptic bouton size in khc20−/− mutants was significantly decreased (p<0.05) while roblk−/− mutants showed an opposite outcome, an increase, although this trend was not significant (Figure 1G, p = 0.064). This difference can be clearly seen in the enlarged immunofluorescence image from roblk−/− mutants where the red area depicting DLC were much larger then the green HRP area (Figure 1B, arrow). In contrast, in khc20−/− mutants all pre synaptic boutons were not surrounded by SSR (Figure 1C).

We further examined the size of the synapse by measuring synaptic length. Synaptic length was measured within the corresponding muscle area to avoid any biases that might arise due to a variation in muscle size among different larvae and genotypes. Interestingly, roblk−/− mutants showed an approximately 4.5 fold decrease (p<0.01) in synaptic length compared to wild type, while khc20−/− mutants showed an opposite result, an increase, although this trend was not significant (Figure 1H, p = 0.33). Taken together these observations suggest that disruption of transport can lead to synaptic morphological defects. Consistent with our results previous work has shown that synaptic function was also disrupted in motor protein mutants; both the action potential propagation in axons and neurotransmitter release were impaired in khc−/− mutants [53] and the EJP amplitudes were reduced in p150glued−/− mutants [54].

Since all synaptic components need to be transported within the axon to nerve terminals, it is commonly rationalized that all synaptic defects must be instigated by defects in axonal transport. To directly test this proposal we evaluated NMJs from larvae that carried a mutation for a synaptic vesicle protein, synaptotagmin (syt). Synaptotagmin is a synaptic vesicle-specific integral membrane protein that has been suggested to play a key role in synaptic vesicle docking and fusion [55]. Synaptotagmin is present within the larval brain, the larval axon, and the pre synapse [56]. It is also transported down the axon [57] via an association with KIF1A, the neuron-specific kinesin 3 motor together with synaptophysin and Rab3A [58] and defects in kinesin-1 or dynein causes syt to accumulate in axonal blocks [63]. Moreover reduction of syt was shown to substantially alter synaptic function at larval NMJs, with decreased neurotransmitter release, smaller evoked synaptic potentials and detectable morphological changes in the arborization of the synapse [59].

**Figure 2:**
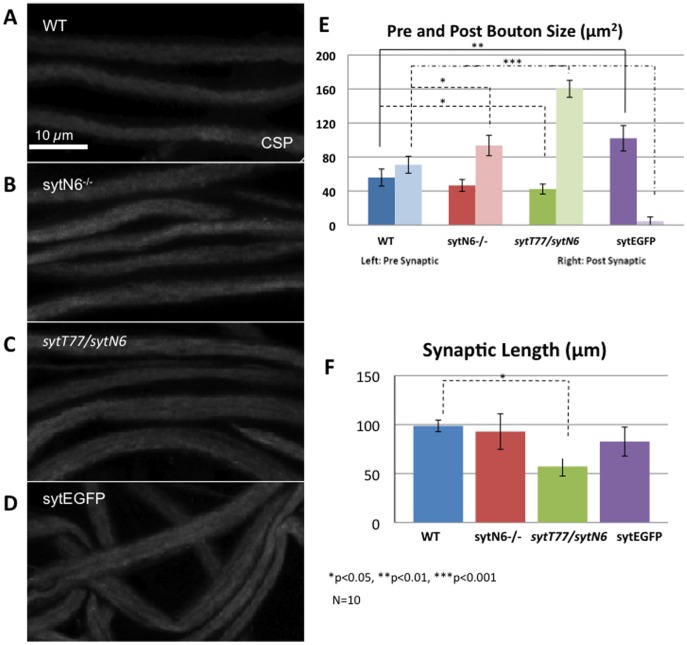
Loss of function of synaptotagmin or gain of function of synaptotagmin does not cause axonal transport defects. **A:** Control wild type (WT) larval segmental nerves show smooth CSP and do not contain axonal blockages. Bar = 20 µm. **B:** Larval segmental nerves from the loss of function mutant of syt, sytN6−/− do not show axonal blockages. **C:** Larval segmental nerves from the synT77/syN6 do not show axonal blockages. **D:** Segmental nerves from larvae expressing syt-EGFP do not show axonal blockages. **E:** Quantitative analysis of the pre and post synaptic bouton areas show significant changes between the pre and post synaptic areas with syt loss of function (synN6−/− post p = 0.0508, sytT77/sytN6 pre = 0.043, post = 0.00087) compared to wild type. Larvae expressing syt-EGFP also show significant changes in their post and pre synaptic areas (pre p = 0.009, post p = 0.00031) compared to wild type. Note that in the syt-EGFP NMJs the pre synaptic bouton area is larger than the post synaptic bouton area. In contrast, sytN6−/− and sytT77/sytN6 mutants show a larger post-synaptic bouton area than the pre-synaptic bouton area. Y axis = average of pre and post synaptic area (µm2). **F:** Synaptic length quantification show significant decreases in the sytT77/sytN6 NMJs (p = 0.005) compared to wild type, similar to roblk−/− NMJs (Figure 1). Y axis = average synaptic length (µm). 10 larvae were imaged and analyzed to evaluate axonal transport and synaptic defects. For statistical analysis ANOVA was used followed by post-hoc analysis using the Bonferroni's test.

To test if these synaptic morphological and functional defects seen in syt loss of function mutant NMJs were due to problems in axonal transport, we examined larval segmental nerves from syt mutants using the cystein string protein (CSP) antibody (Figure 2). Many of the syt null mutations were lethal at early stages, viable only to the 1^st^ instar larval stage [60], [61] and were too small to dissect. To obtain 3^rd^ instar syt mutant larvae, we used a culturing method where food was consistent along with high humidity which previously permitted the survival of the amorphic sytAD4 mutant through early adulthood [62]. Third instar sytAD4 mutant larvae from these cultures exhibited severely decreased and asynchronous evoked neurotransmitter release, as well as an increased rate of spontaneous neurotransmitter release [62]. Using this culturing technique hypermorphic sytT77 and amorphic sytN6 larvae survived to late 3^rd^ instar and some pupated, in contrast to normal culturing conditions where both these mutants were lethal at early larval stages. We also cultured and examined 3^rd^ instar larvae that were sytT77/sytN6. Larval segmental nerves from homozygous sytT77−/−, homozygous sytN6−/− and heterozygous sytT77/sytN6 did not show axonal blockages in contrast to larvae that were kinesin-1−/− or dynein−/− (Figure 2BC, Figure 1). However, all syt mutant larvae showed synaptic morphological defects; defects in pre and post synaptic bouton size and synaptic length (Figure 2EF). Significant changes in the post synaptic bouton size and in the ratio of the pre and post synaptic bouton size was seen in heteroallelic sytT77/sytN6 (p<0.05) and homozygous syntN6−/− larvae (Fig. 2E). Synaptic length was also significantly decreased (p<0.05) in heteroallelic sytT77/sytN6 larvae (Figure 2F). Thus the synaptic morphological and functional defects seen in syt mutant larvae were not the consequence of axonal transport defects, but rather the result of syt loss of function. Since syt is present in axonal blockages observed in motor protein mutants [63], the synaptic morphological defects observed in motor protein mutants likely resulted from disruption of transport to NMJs. Therefore while perturbations in axonal transport can cause synaptic defects, synaptic problems do not cause axonal transport defects. Thus a direct downstream consequence of axonal transport defects likely is synapse dysfunction.

We rationalized that if problems in axonal transport occurred early, and over time resulted in morphological and functional defects at the synapse then we should observe axonal blockages or accumulations early during development, earlier than the larval stages. To test this we examined embryonic axons in motor protein mutants. Embryos were collected, fixed and stained with antibodies against CSP. Using high resolution confocal microscopy, axonal accumulations in embryonic axons were observed in stage 15–17 embryos carrying the homozygous loss of function dynein mutant (roblk−/−), while wild type embryonic axons stained smoothly (Figure S2). Homozygous roblk−/− embryos were identified using the Tubby marker, as stage 15–17 embryos carrying the Tb gene were much shorter and fatter than non-Tubby homozygous roblk−/− embryos. Our observations suggest that axonal problems can occur early during development, which over time likely contributes to the synaptic abnormalities, the developmental defects and organismal lethality observed in roblk−/−.

### Transgenic lines expressing human disease proteins that show axonal transport defects also show abnormalities in synaptic morphology

**Figure 3:**
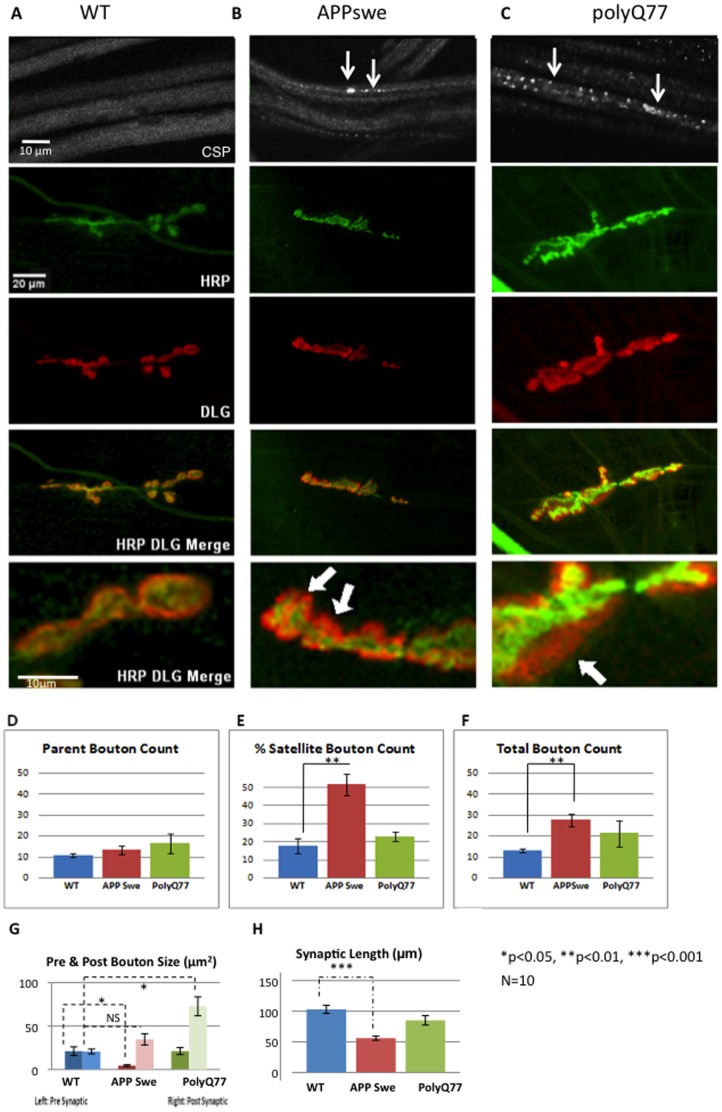
Transgenic lines expressing human disease proteins that show axonal transport defects also show abnormalities in synaptic morphology. **A:** Larval segmental nerves from wild type larvae show smooth staining with the synaptic vesicle marker CSP. Pre (HRP-green) or post synapses (DLG-red) are clearly observed in these larval NMJs. Bar = 10 µm and 20 µm. **B:** Segmental nerves from larvae expressing human APPswe show axonal blockages similar to previous observations (arrows) [63]. Both pre (HRP, green) and post synaptic markers (DLG, red) show synaptic morphology defects. Note that the post synaptic area (red) is much greater than what is observed in wild type in the enlarged image (arrow). **C:** Segmental nerves from larvae expressing pathogenic polyQ77 show axonal blockages similar to previous observations (arrows) [97]. Pre (HRP, green) and post synaptic (DLG, red) markers show synaptic morphology defects. Note that the post synaptic areas (red) is much great than what is observed in wild type (arrow) in the enlarged image. **D:** Quantitative analysis did not show any significant changes in the average number of parent boutons in larvae expressing APPswe (p = 0.320) or polyQ77 (p = 0.326) compared to wild type. Y axis is the average number of parent boutons. **E:** Quantitative analysis of the percent of satellite boutons shows a significant increase in larvae expressing APPswe compared to wild type (p = 0.0069), while larvae expressing polyQ77 are similar to wild type (p = 0.193). Y axis is the percent of satellite boutons. **F:** The total number of boutons are significantly increased in larvae expressing APPswe (p = 0.0101), while no significant change is seen in larvae expressing polyQ77 compared to wild type (p = 0.277). The increase in the total number of boutons in larvae expressing APPswe is the result of an increase in the number of satellite boutons. Y axis is the total number of boutons. **G:** Quantitative analysis of synaptic bouton area reveals a significant reduction in the pre-synaptic bouton areas of APPswe larvae (p = 0.0468) compared to wild type. These defects are similar to what was seen in khc−/− larvae (Figure 1G). The pre synaptic bouton areas from larvae expressing polyQ77 are similar to wild type (p = 0.9886). Y axis is the pre synaptic area µm2. While a significant reduction is not observed in the post synaptic bouton areas in APPswe larvae (p = 0.12), a significant increase in the post synaptic boutons areas is seen in polyQ77 (p = 0.018) larvae compared to wild type. Y axis is the pre synaptic area µm2. H: A significant decrease is seen in the synaptic length in APPswe larvae (p = 0.00024), similar to roblk−/− NMJs (Figure 1), while polyQ77 larvae are similar to wild type (p = 0.133). Y axis = average synaptic length (µm). 10 larvae were imaged and analyzed to evaluate axonal transport and synaptic defects. For statistical analysis ANOVA was used followed by post-hoc analysis using the Bonferroni's test.

Many human neurodegenerative diseases, including Alzheimer's disease (AD) and Huntington's (HD)/other poly Q diseases display axonal pathologies including axonal blockages and abnormal accumulations of proteins and organelles. Growing evidence supports the proposal that defects in axonal transport could contribute to the pathogenesis observed in these neurodegenerative diseases [64]. Included in this neuropathology are synaptic loss, dysfunction and abnormal transmission. We previously showed that larvae expressing human amyloid precursor protein with the Swedish mutation (APPswe) contained axonal transport defects [63] (Figure 3AB). Larvae expressing expanded amounts of glutatmine repeats (polyQ77) also showed axonal transport defects [97] (Figure 3AC). However, whether these larvae also contained synaptic morphological abnormalities were not examined. To test whether transport defects induced by APPswe or pathogenic polyQ77 also caused synaptic abnormalities, the NMJs of these larvae were evaluated. Type 1 synaptic boutons between muscle 6 and 7 at larval abdominal segments A4–5 were examined with pre and post synaptic markers (HRP and DLG, Figure 3AB). As controls, wild type (YW), APPL-GAL4 and UAS-APPswe/polyQ lines were examined and were undistinguishable from each other. Strikingly, a significant increase in the number of satellite boutons was observed in larvae expressing APPswe compared to wild type (2.5 fold, p<0.01, Figure 3E). A 1.5 fold increase (p<0.01, Figure 3F) was seen in the total bouton number in these larvae. This increase in the total number of boutons was due to the number of satellite boutons and was not the result of an increase in the number of non satellite boutons of normal appearance (parent boutons, Figure 3D). The total number of boutons included the number of satellite boutons and the number of parent boutons at muscles 6 and 7 (Figure 3F). These results were consistent with previous observations in larvae expressing Drosophila amyloid precursor-like (APPL) protein [51]. An ∼2.5-fold increase in the total number of boutons, and a 3 fold increase in the percentage of satellite boutons was observed in larvae expressing APPL compared to control larvae [51]. In contrast, no significant changes were seen in the number of parent boutons (p = 0.326) or in the percentage of satellite boutons for larvae expressing pathogenic polyQ repeats (polyQ77, p = 0.193).

Further examination of synaptic morphology indicated that the pre synaptic bouton size was significantly reduced in larvae expressing APPswe, while the post synaptic bouton size was not (Figure 3G). This 1.5 fold decrease in the pre synaptic bouton size was responsible for the significant change observed for the ratio of the pre and post synapse (p<0.05). In contrast, while no significant changes were observed for the pre synapse in larvae expressing pathogenic polyQ repeats (p = 0.9886), a 5 fold increase was seen for the post synapse (p<0.01) compared to control larvae. This 5 fold increase in post synaptic bouton size was responsible for the significant change observed for the ratio of the pre and post synapse (p<0.01, Figure 3G). Enlarged immunofluorescence images clearly show that the post synapse (red) was much larger in size than the pre synapse (green) compared to controls (Figure 3ABC, arrows). Strikingly these changes were similar to what was observed for dynein mutants (roblk−/−, Figure 1AB), indicating that these morphological changes could result as a consequence of defects in axonal transport.

Further, synaptic length was also significantly decreased (p<0.005) in larvae expressing APPswe, but no significant change was seen in larvae expressing pathogenic polyQ77 compared to control larvae (Figure 3H, p = 0.133). The synaptic decrease observed in APPswe NMJs was approximately 4.2 fold compared to wild type. Interestingly, this reduction in synaptic length was strikingly similar to the decreases in synaptic length seen in dynein mutants (roblk−/− Figure 1H).

To evaluate whether the synaptic defects we observed in larvae expressing APPswe and pathogenic polyQ77 were due to transport defects we evaluated larvae expressing the synaptic protein synaptotagmin. While larval segmental nerves in synaptotagmin expressing larvae did not contain axonal blockages (Figure 2D), striking synaptic morphological defects were observed (Figure 2E). While syt expressing larval nerves showed smooth CSP staining comparable to wild type nerves, the NMJs of these larvae showed increases in pre synaptic bouton size and decreases in post synaptic bouton size (Figure 2E). While a significant increase was seen in the pre synaptic bouton size (p<0.01), a significant decrease was seen in the post synaptic bouton size was (p<0.001, Figure 2E). A 6 fold decrease was observed, which lead to a significant change in the ratio of the pre and post synaptic bouton size (Figure 2E). Strikingly, these results were opposite to what was observed for the loss of function syt mutants (Figure 2E) indicating that these phenotypes were a direct consequence of syt function at the NMJs. Since syt accumulations were observed in APPswe and pathogenic polyQ77 larval nerves [63], [97], collectively, these results suggest that the synaptic morphological abnormalities observed in these disease protein expressing larvae likely resulted from perturbations in axonal transport of essential components to the NMJs.

### BMP signaling, as measured by its downstream signal, phospho Mad is decreased in motor protein mutants

**Figure 4:**
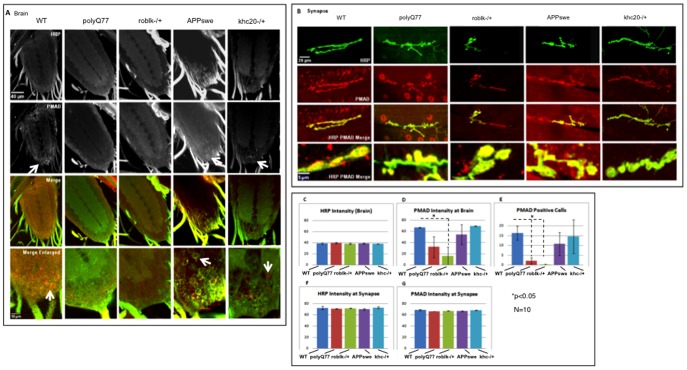
BMP signaling, as measured by its downstream signal, phospho Mad is decreased in motor protein mutants. The BMP signaling was measured using phospho MAD (p-Mad) in motor neuron cell bodies located in the larval brain (**A**) and in larval NMJs (**B**). The pre-synaptic marker HRP was used as a control. **A:** In wild type brains p-Mad is seen in motor neuron cell body nuclei (arrow in enlarged image). Strikingly, p-Mad is absent in roblk−/− motor neuron cell body nuclei. Decrease in p-Mad is also seen in cell body nuclei from larval brains expressing polyQ. No change in HRP is seen in these larval brains. p-Mad positive cell body nuclei are also seen in khc20−/− and larvae expressing APPswe (arrow). Bar = 40 µm and 10 µm (enlarged) **B:** No striking changes are observed in p-Mad or HRP at NMJs from wild type, roblk−/−, khc20−/−, APPswe or polyQ77 larvae. **C:** Quantitative analysis of HRP intensity in larval brains from wild type, rolbk−/−, khc20−/−, APPswe and polyQ77 show no changes in HRP intensity. Y axis represents percent intensity. **D:** In contrast, quantitative analysis of p-Mad intensity in roblk−/− larval brains show a significant decrease compared to wild type (p = 0.0404), while a decreasing trend is seen in larval brains expressing polyQ77, but this is not significant (p = 0.08). Both khc20−/− (p = 0.156) and APPswe (p = 0.53) did not show any significant change. Y axis represents percent intensity. **E:** Quantitative analysis of p-Mad positive cells show significant reductions in p-Mad positive cells in roblk−/− (p = 0.0201), and in polyQ77 larval brains (p = 0.0239), compared to wild type. Although reductions are also seen in khc−/− (p = 0.58) and APPswe (p = 0.46) brains this trend is not significant. Y axis represents the average number of p-Mad positive cells. **F:** No changes are observed in HRP intensity at the NMJs for all of these genotypes. Bar = 20 µm and 5 µm enlarged. **G:** No changes are observed in p-Mad intensity at the NMJs for all genotypes. N = 10 larvae.

In Drosophila, BMP signaling has been shown to regulate synaptic growth, function and stabilization at the NMJ [10], [38]–[40], [65]. Recently, a role for BMP signaling was also shown in axonal transport [43]. Therefore we tested the hypothesis that the synaptic morphological abnormalities that we observed in motor protein mutants could result due to the inhibition or down regulation of BMP signaling. Previous work found that BMP signaling was altered in the motor neurons of stage 17 embryos expressing a truncated form of p150/Glued, a component of the dynactin complex, leading to the proposal that problems in transport interferes with BMP signaling [10]. Similarly, we also found that BMP signaling as assayed by its downstream signal phospho Mad (p-Mad) was decreased in the ventral ganglion motor neuron cell bodies of larvae carrying the dynein mutation, roblk−/−, while larvae carrying the kinesin mutation, khc20−/− were comparable to wild type larvae (Figure 4).

It has been proposed that the BMP ligand Glass Bottom Boat (Gbb) provides a retrograde signal from the muscle to the nerve terminal. Receptor activation then leads to an increase in the phosphorylation of R-Smad, Mad at the NMJ terminals followed by nuclear translocation of p-Mad in the brain through its interaction with the co-Smad, Medea (med) [9]. Nuclear translocation of p-Mad is proposed to occur via the retrograde transport of p-Mad- containing signaling complexes [66], [40], [10]. However, recent work has also proposed that two distinct populations of p-Mad exists; one at the synapse and one at the cell body [67] suggesting that p-Mad signaling may not occur via a retrograde signaling endosome. To evaluate BMP-signaling in the context of axonal transport we quantified the p-Mad levels in motor protein mutants using two methods, 1) by measuring the intensity of the p-Mad staining within the ventral ganglion, and 2) by counting the number of p-Mad positive cells in the ventral ganglion. Both methods showed a significant decrease in the intensity of p-Mad and in the number of p-Mad positive cells in the ventral gangion for roblk−/− mutant larvae compared to wild type (Figure 4ADE). A 4 fold decrease in p-Mad intensity (p<0.01) was seen in roblk−/− brains compared to wild type brains (Figure 4D), while a 15 fold decrease (p<0.001) was seen in the number of p-Mad positive cells in roblk−/− brains compared to wild type brains (Figure 4E). These decreased p-Mad levels were rescued by expression of roblk (data not shown) suggesting that the reduction in p-Mad levels at the cell bodies was a direct result of loss of dynein motors. Further, while a trend towards a decrease in p-Mad positive cells were seen in khc20−/− brains, this change was not significant. In contrast no significant change in the intensity of HRP was seen in roblk−/−, khc20−/− or wild type larval brains (Figure 4AC).

To further evaluate whether the observed changes in p-Mad levels were due to changes in p-Mad at the NMJ we quantified the intensity of p-Mad at NMJs. Surprisingly, no significant change in p-Mad intensities were seen at NMJs from roblk−/− or khc20−/− larvae (Figure 4BG). The NMJ p-Mad intensities in both these mutants were comparable to the p-Mad intensities seen in WT NMJs. No significant change in the level of HRP intensities was seen at NMJs from roblk−/−, khc20−/− or wild type larvae (Figure 4F). Collectively these results suggest that the retrograde transport of p-Mad was altered in roblk−/− mutants and that this defect was not the consequence of reductions in p-Mad levels at NMJs. Thus, the retrograde motor dynein appears to be directly responsible for the retrograde transport of p-Mad to larval cell bodies located in the brain.

**Figure 5:**
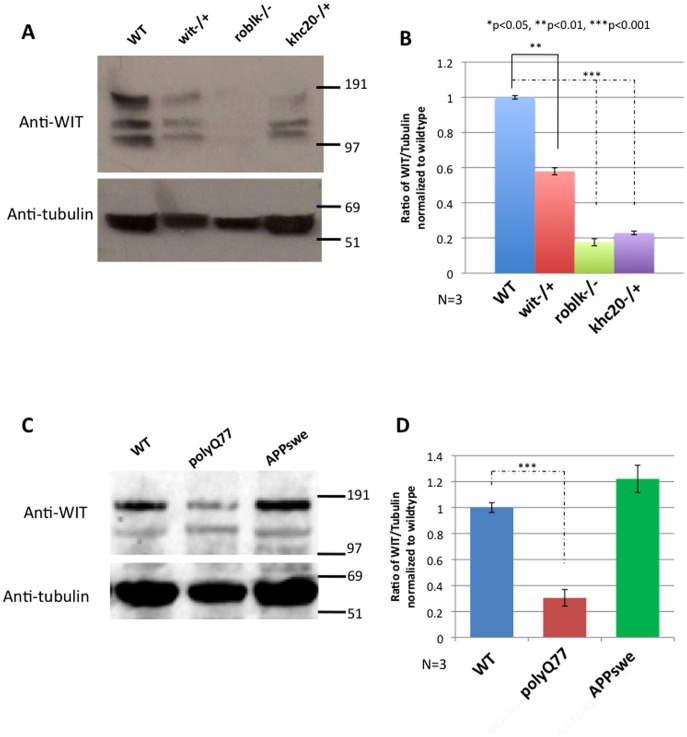
The level of wishful thinking (WIT) protein, a component of the BMP pathway is decreased in motor protein mutants. Biochemical analysis of the total amount of protein is assayed in wild type, roblk−/−, khc20−/+ and wit−/+ brains. **A:** A representative gel shows that the total level of WIT protein is significantly reduced in roblk−/− and khc20−/+ larval brains compared to wild type larval brains. Reductions are also seen in wit−/+ control brains compared to wild type. Tubulin is used as a loading control. **B:** Quantitative analysis from 3 independent experiments show that the ratio of WIT protein is significantly decreased in roblk−/− (p = 0.0001), khc20−/+ (p = 0.0003) and wit−/+ (P = 0.007) compared to wild type. The ratio of wit was compared to tubulin and normalized to wild type. **C:** A representative gel shows that the total level of WIT protein is significantly reduced in polyQ77 larval brains compared to wild type larval brains, but not in APPswe larval brains. Tubulin is used as a loading control. **D:** Quantitative analysis of three blots show that the ratio of WIT protein is significantly decreased in polyQ77 larval brains (p = 0.0004) compared to wild type. No significant change was seen in APPswe larval brains.

We further probed how the BMP signaling pathway was affected in motor protein mutants by evaluating the level of Wishful thinking (wit). Wit, a type II BMP receptor mediates signaling via ligand binding, recruitment and phosphsorylation of type 1 receptors resulting in the phosphorylation of p-Mad to regulate NMJ synapse development [68], [38], [40]. Using biochemical analysis we found that the level of WIT protein was decreased in motor protein mutant larvae. The total level of WIT was greatly reduced in both the roblk−/− and khc20−/+ larval brains compared to wild type. Reductions were also seen in wit−/+ larval brains indicating the specificity of the WIT antibody (Figure 5A). Quantification analysis revealed that these reductions in WIT protein levels were significant (Figure 5B). The level of WIT was depicted as a ratio of the level of TUBULIN and normalized to the ratio of WIT/TUBLIN for wild type larval brains, which was set at 1. Roblk−/− showed a 5.7 fold decrease, while khc20−/+ showed a 4.5 fold decrease in WIT levels compared to wild type. Wit−/+ showed a 1.75 fold decrease in WIT protein. Our results suggest that while defects in transport disrupts the trafficking of BMP components to the synapse, p-Mad activation at the NMJs was not dramatically perturbed, but retrograde p-Mad signaling to the cell bodies was disrupted. Therefore while defects in transport likely decreases the relative amount of BMP components to the synapse, transport defects do not directly influence the distribution of BMP components within the nerve terminals or the activation of BMP signaling at the NMJs.

### BMP signaling, as measured by its downstream signal, phospho Mad is decreased in larvae expressing pathogenic polyQ protein

Since both axonal transport defects and synaptic morphological abnormalities were observed in transgenic lines expressing APPswe and pathogenic polyQ77, we also tested whether retrograde transport of BMP signaling was altered in these larval brains. Using the p-Mad antibody and the quantification methods previously used, changes in the level of p-Mad was observed in larvae expressing pathogenic polyQ77 protein. While quantification of p-Mad intensity did not show a statistically significant decrease (perhaps due to the variation between different larvae) (Figure 4AD), a significant decrease (approximately 10 fold) was seen in the number of p-Mad positive cells in pathogenic polyQ77 larval brains compared to wild type (p<0.001) (Figure 4AE). No change was seen in HRP (Figure 4AC). Further, no change was seen for both p-Mad and HRP intensities at the NMJs of pathogenic polyQ77 larvae (Figure 4BFG). Interestingly, the total level of WIT was greatly reduced in larval brains expressing pathogenic polyQ77 compared to wild type in Western blot analysis (Figure 5C). Quantification revealed that these reductions in WIT protein levels with pathogenic polyQ were significant (Figure 5D). The level of WIT was depicted as a ratio of the level of TUBULIN and normalized to the ratio of WIT/TUBLIN for wild type larval brains, which was set at 1. Pathogenic polyQ77 expressing larvae showed a 4.2 fold decrease in WIT levels compared to wild type. Strikingly, these observations were similar to what was seen in roblk−/−, and down regulation of BMP signaling likely contributed to the synaptic morphological abnormalities observed in pathogenic polyQ77 larvae. In contrast, larvae expressing APPswe did not show significant changes in the level of p-Mad; both in p-Mad intensity and in the number of p-Mad positive cells (Figure 4DE). No significant changes in the total level of WIT was seen in larvae expressing APPswe (Figure 5CD). Perhaps pathogenic polyQ may severely perturb retrograde transport, similar to what was observed in roblk−/−.

### Components of the BMP signaling pathway genetically interact with motor proteins

Similar to neurotrophin signaling, neuronal BMP signaling is proposed to require intact retrograde axonal transport [11], [39], [40], [10]. Inhibition of dynein blocked BMP signaling as assayed by p-Mad indicating that activated BMP components, perhaps in a signaling endosome similar to NGF-TrkA must be retrogradely trafficked along the axon from the synaptic terminal to the cell body. To test this possibility we tested whether components of the BMP pathway and molecular motor proteins showed functional interactions with each other.

**Figure 6:**
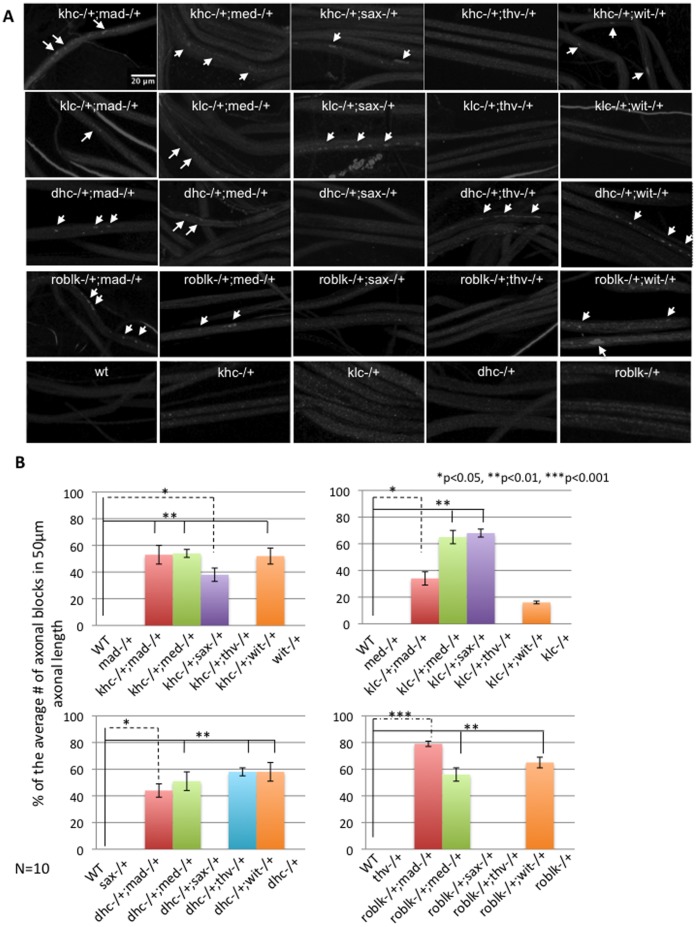
Components of the BMP signaling pathway genetically interact with motor proteins. **A:** Larval segmental nerves from heteroallelic combinations of kinesin or dynein mutations with mutations in the BMP signaling pathway were examined using CSP. Khc−/+, klc−/+, dhc−/+, roblk−/+, mad−/+, med−/+, sax−/+, thv−/+ and wit−/+ were comparable to wild type and their larval nerves showed smooth staining. In contrast axonal blockages are observed in khc−/+;mad−/+, klc−/+;mad−/+, dhc−/+;mad−/+ and roblk−/+;mad−/+ (arrows). No blockages are seen in mad−/+, khc−/+, klc−/+, dhc−/+ and roblk−/+. Axonal blockages (arrows) are seen in khc−/+;med−/+, klc−/+;med−/+, dhc−/+;med−/+ and roblk−/+;med−/+. No blockages are seen in med−/+. Axonal blockages are seen in khc−/+;sax−/+ and klc−/+;sax−/+ (arrows) but not in dhc−/+;sax−/+ and roblk−/+;sax−/+. Axonal blockages are seen in dhc−/+;thv−/+ (arrows), but not in khc−/+;thv−/+, klc−/+;thv−/+ and roblk−/+;thv−/+. Axonal blockages are also seen in khc−/+;wit−/+, dhc−/+;wit−/+ and roblk−/+;wit−/+ (arrows) but not klc−/+;wit−/+. Bar = 10 µm. **B:** Quantification analysis of the % of the average number of axonal blockages in 50 µm nerve length show significant amounts of axonal blockages in khc−/+;mad−/+,khc−/+;med−/+, khc−/+;sax−/+, khc−/+;wit−/+, klc−/+;mad−/+, klc−/+;med−/+, klc−/+;sax−/+, dhc−/+;mad−/+, dhc−/+;med−/+, dhc−/+;thv−/+, dhc−/+;wit−/+, roblk−/+;mad−/+, roblk−/+;med−/+, and roblk−/+;wit−/+, compared to wild type, khc−/+, klc−/+, dhc−/+, roblk−/+, mad−/+, med−/+, sax−/+, thv−/+ or wit−/+. N = 10 larvae. For statistical analysis ANOVA was used followed by post-hoc analysis using the Bonferroni's test.

Drosophila larvae carrying loss of function mutations of kinesin-1 (khc, klc) and dynein (dhc, roblk) show a distinct axonal accumulation phenotype [44]–[46]. Segmental nerves from these mutant larvae show accumulations of synaptic proteins such as cystein string protein (CSP, Figure 1 top panel). Similar to these phenotypes, previous work has shown that loss of function mutations of proteins functioning in the BMP signaling pathway also showed axonal accumulations [38], [43], [69], but not to the same extent as mutations of motor proteins. To test the hypothesis that components in the BMP pathway are transported within axons by associating with molecular motors, we performed genetic interaction tests between BMP proteins and motor proteins. Heteroallelic combinations of BMP receptors (wit, thv and sax), BMP ligands (mad and med), with kinesin-1 (khc, klc) or dynein (dhc, roblk) were generated as previously done [63], [97]. Larvae carrying heteroallelic combinations were dissected and assayed for axonal transport defects. As controls, mad−/+, med−/+, sax−/+, thv−/+, wit−/+, khc−/+, klc−/+, dhc−/+, roblk−/+ larvae were assayed and all these larvae were comparable to wild type showing smooth CSP staining in their larval segmental nerves (Figure 6). In contrast, many of the heteroallelic BMP-motor protein combinations showed axonal blockages (Figure 6). Heteroallelic larvae carrying mutations of mad and khc (mad−/+;khc−/+), mad and klc (mad−/+;klc−/+), mad and dhc (mad−/+;dhc−/+) or mad and roblk (mad−/+;roblk−/+) all showed significant amounts of axonal blockages, indicating that MAD uses both kinesin-1 and dynein for bi-directional movement within axons (Figure 6). Heteroallelic larvae carrying mutations of med and khc (med−/+;khc−/+), med and klc (med−/+;klc−/+), med and dhc (med−/+;dhc−/+) and med and roblk (med−/+;roblk−/+) all showed significant axonal blockages, indicating that MED also uses kinesin-1 and dynein for bi-directional movement within axons (Figure 6). However only heteroallelic larvae that were carrying mutations of sax and khc or klc (sax−/+;khc−/+ and sax−/+;klc−/+) showed axonal blockages, while larvae that were sax−/+;dhc−/+ and sax−/+;roblk−/+ did not show axonal blockages (Figure 6). While SAX may be transported to the nerve terminal via kinsein-1 perhaps SAX undergoes degradation at nerve terminals and is not returned back to the soma. Larvae that were wit−/+;dhc−/+, wit−/+;roblk−/+ and wit−/+;khc−/+ showed axonal blockages, while larvae that were wit−/+;klc−/+ did not (Figure 6). Only tkv−/+;dhc−/+ larvae showed axonal blockages (Figure 6). Our results are consistent with recent observations suggesting that WIT, TKV and MAD move bi-directionally within axons [67].

**Figure 7:**
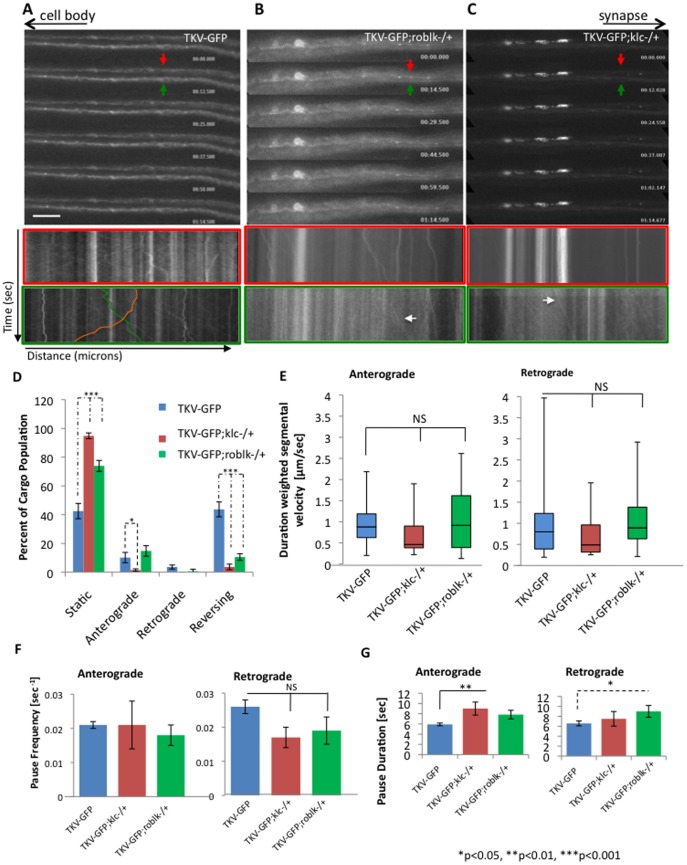
Reduction of motor proteins perturbs the bi-directional movement of TKV vesicles. **A:** TKV-GFP vesicles move bi-directionally in larval axons. Cell bodies are towards the left and the synapse is towards the right as depicted by the arrows at the top. Representative kymographs depicts the movement of TKV-GFP. Both anterograde (green) and retrograde (orange) TKV vesicles are observed. X axis = distance (µicrons) and Y axis = time (sec). Bar = 20 µm. The kymograph outlined in red is from the top axon (red arrow), while the kymograph outlined in green is from the bottom axon (green arrow). **B:** Reduction of dynein (TKV-GFP;roblk−/+) perturbs the movement of TKV-GFP and GFP blockages can be observed. Note that the representative kymographs show large stalled GFP blockages and slow moving TKV-GFP vesicles (white arrows). **C:** Reduction of kinesin (TKV-GFP;klc−/+) also perturbs the movement of TKV-GFP and GFP blockages can be observed. Note that while the kymograph from the top axon (red arrow) shows large stalled GFP blockages, the kymograph from the bottom axon (green arrow) shows slow moving TKV-GFP vesicles (white arrow). For each genotype, a total of 5 animals were imaged for motility data analysis; four time-lapsed movies were collected for each animal; a total of 20 movies were analyzed using our custom particle tracking program. **D:** Percentage of cargo populations indicate a significant increase in the percent of stalled or static cargo for TKV-GFP;klc−/+ (p = 8.66×10^−14^) or TKV-GFP;roblk−/+ (p = 3.49×10^−8^) larvae compared to TKV-GFP larvae with 100% kinesin or dynein. Significant decreases in the % of anterograde TKV-GFP vesicles (p = 0.048) were observed in TKV-GFP;klc−/+ larvae compared to TKV-GFP larvae. Significant decreases in the % of reversing TKV-GFP vesicles were observed in TKV-GFP;klc−/+ (p = 1.35×10^−11^) or TKV-GFP;roblk−/+ (p = 1.41×10^−9^) larvae compared to TKV-GFP larvae. Table 1 shows the number of vesicles for each genotype. (TKV-GFP = 184 vesicles, TKV-GFP;klc−/+ = 136 vesicles, TKV-GFP;roblk−/+ = 142 vesicles). Note that 50% reduction of motors caused most of the TKV-GFP vesicles to stall and only very few vesicles showed motility. (TKV-GFP;klc−/+ anterograde = 2 vesicles, retrograde = 0 vesicles, reversing = 5 vesicles, TKV-GFP;roblk−/+ anterograde = 21 vesicles, retrograde = 1 vesicle, reversing = 15 vesicles, compared to TKV-GFP anterograde = 17 vesicles, retrograde = 6 vesicles, reversing  = 73 vesicles). Arrows indicate the direction of change with 50% reduction of motors. **E:** Box plots of duration-weighted segmental velocities of TKV-GFP vesicles in larvae with 50% kinesin or dynein compared to larvae with 100% kinesin or dynein show no significant changes (NS) in both the anterograde and retrograde velocities. Box plots outline the distribution of duration-weighted segmental velocities for each genotype. The horizontal bar represents the median. The upper and lower box edges represents 75% percentile (i.e. upper quartile) and 25% percentile (i.e. lower quartile), respectively. Note that motility analysis was calculated from net anterograde and retrograde moving vesicles and reversing vesicles. NS = not significant. **F:** No significant changes in pause frequencies were observed for anterograde or retrograde velocities in TKV-GFP;klc−/+ or TKV-GFP;roblk−/+ larvae compared to TKV-GFP larvae. **G:** However, significant increases in anterograde pause durations were seen for anterograde vesicles in TKV-GFP;klc−/+ (p = 0.006) larvae compared to TKV-GFP larvae. Significant increases in retrograde pause durations were also observed in TKV-roblk−/+ (p = 0.014) larvae compared to TKV-GFP larvae. Table 2 shows the summary of the *in vivo* measurements obtained from genotypes TKV-GFP, TKV-GFP;klc−/+ and TKV-GFP;roblk−/+.

We further evaluated whether reductions in molecular motors have a functional influence on the movement of BMP components within axons by using *in vivo* motility analysis. Larval segmental axons expressing TKV-GFP in the context of roblk−/+ or klc−/+ were imaged as previously done [57]. Within larval axons TKV-GFP vesicles showed bi-directional movement (Figure 7A), similar to SYNT-EGFP or APP-YFP vesicles [57]. The average anterograde and retrograde duration weighted segmental velocities of TKV vesicles were 0.9466 and 0.9982 microns/sec respectively (Figure 7E), similar to the velocity rates observed for SYNT-EGFP or APP-YFP vesicles [57]. Strikingly, 50% reduction of kinesin (klc−/+) or dynein (roblk−/+) in the context of larvae expressing TKV-GFP perturbed the motility of TKV vesicles and caused TKV to accumulate into large GPF blockages (Figure 7A–C, Movie S1, S2, S3). Quantification analysis of movement dynamics showed significant decreases in TKV motility with 50% reduction of motors compared to 100% motors. Cargo population analysis showed significant increases in the population of stalled or static TKV vesicles with 50% reduction of klc−/+ (p = 8.66×10^−14^) or roblk−/+ (p = 3.49×10^−8^) compared to TKV with 100% motors (Figure 7D, Table 1). Interestingly, only a few TKV vesicles were moving in the net anterograde or retrograde directions or reversing in the context of 50% reduction of motors in contrast to TKV with 100% motors (Figure 7D, 7A–C kymographs arrows). While no significant changes in velocities were seen in the few anterogradely or retrogradely moving TKV vesicles in the context of 50% motors, the pause duration of these vesicles were significantly increased (Figure 7GH, anterograde = p = 0.006, retrograde = p = 0.014, Table 2) indicating that reduction of motors perturbs the motility of TKV vesicles. Thus, the BMP receptor TKV likely associates with molecular motors kinesin-1 and dynein for bi-directional movement within axons (Figure S3).

**Table 1:** Summary of the number of vesicles for genotypes TKV-GFP, TKV-GFP;klc−/+ and TKV−GFP;roblk−/+.

	TKV-GFP	TKV-GFP;klc−/+	TKV-GFP;roblk−/+
Total number of vesicles	184	136	142
Stationary vesicles	71	129	105
		p = 8.66×10^−14^ ***	p = 3.49×10^−8^ ***
Anterograde vesicles	17	2	21
		p = 0.048*	
Retrograde vesicles	6	0	1
Reversing vesicles	73	5	15
		p = 1.35×10^−11^ ***	p = 1.41×10^−9^ ***

*Significance <0.05,

**significance <0.01,

***significance <0.001 as determined one way ANOVA followed by Bonferroni's test.

**Table 2:** Summary of *in vivo* measurements from the customized single particle tracking software program obtained from genotypes TKV-GFP, TKV-GFP;klc−/+ and TKV-GFP;roblk−/+.

	TKV-GFP	TKV-GFP;KLC−/+	TKV-GFP,roblk−/+
Anterograde duration- weighted segmental velocity (mean±SEM; µm/sec.)	0.9466±0.0474	0.7957±0.2857	1.0162±0.1164
Retrograde duration-weighted segmental velocity (mean ±SEM; µm/sec.)	0.9982±0.0926	0.7963±0.3156	1.0680±0.1881
Anterograde segmental pause frequency (mean±SEM; pause/sec.)	0.021±0.001	0.021±0.007	0.018± 0.003
Retrograde segmental pause frequency (mean ±SEM; pause/sec.)	0.026±0.002	0.017±0.003	0.019±0.004
Anterograde pause duration (mean ±SEM; sec.)	5.908±0.263	9±1.278	7.825±0.843
		p = 0.006**	
Retrograde pause duration (mean ± SEM; sec.)	6.581±0.491	7.5±1.475	9±1.188
			p = 0.014*

Arrows indicate direction of change with 50% reduction of motors.

*Significance <0.05,

**significance <0.01,

***significance <0.001 as determined by nonparametric Wilcoxon-Mann-Whitney test. Distributions were determined to be non-normal by Lilliefors test.

**Figure 8:**
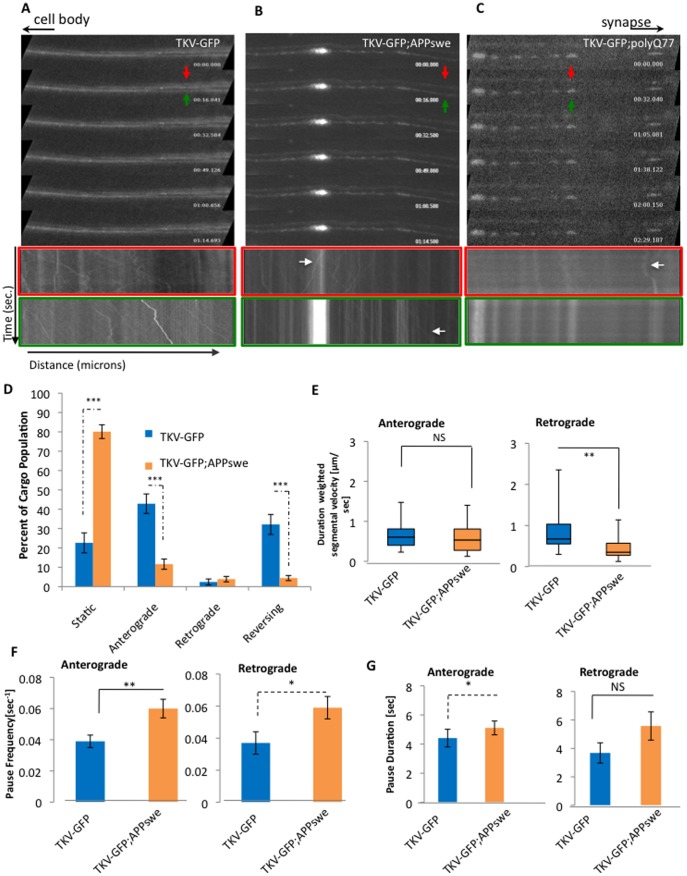
APPswe and PolyQ77 perturb the bi-directional movement of TKV vesicles. **A–C:** While TKV-GFP vesicles move bi-directionally in larval axons, expression of APPswe or polyQ77 perturbs the movement of TKV-GFP and GFP blockages can be observed. Representative kymographs from APPswe and polyQ77 show large stalled blockages of TKV-GFP with decreased movement of TKV-GFP. The kymograph outlined in red is from the top axon (red arrow), while the kymograph outlined in green is from the bottom axon (green arrow). Note that while some decreased TKV vesicle motility can be observed in APPswe (white arrow), almost all of the TKV vesicles are stalled in polyQ77. TKV-GFP was very dim in polyQ77 axons compared to TKV-GFP or TKV-GFP;APPswe larval axons, presumably due to the accumulation of TKV-GFP in cell bodies (data not shown). X axis = distance (µicrons) and Y axis = time (sec). Cell bodies are towards the left and the synapse is towards the right as depicted by the arrows at the top. A total of 5 animals were imaged for motility data analysis; four time-lapsed movies were collected for each animal; a total of 20 movies were analyzed using our custom particle tracking program. These movies were obtained at 2000 msec. **D:** Percentage of cargo populations indicate a significant increase in the percent of stalled or static cargo for TKV-GFP;APPswe larvae compared to TKV-GFP larvae alone (p = 6.64×10^−11^). Significant decreases in the % of anterograde (p = 4.75×10^−6^) and in the % of reversing cargo (p = 5.05×10^−6^) were observed in TKV-GFP;APPswe compared to TKV-GFP larvae. Table 3 shows the number of vesicles for each genotype. (TKV-GFP = 184 vesicles, TKV-GFP;APPswe = 181 vesicles). Note that expression of APPswe causes most of the TKV-GFP vesicles to stall and only very few vesicles show motility. (TKV-GFP;APPswe anterograde = 21 vesicles, retrograde = 7 vesicles, reversing = 8 vesicles, compared to TKV-GFP anterograde = 83 vesicles, retrograde = 2 vesicles, reversing = 63 vesicles). Arrows indicate the direction of change for TKV-GFP;APPswe compared to TKV-GFP. **E:** Box plots of duration-weighted segmental velocities of TKV-GFP vesicles in larvae expressing APPswe show a significant decrease in retrograde velocities of TKV-GFP vesicle movement (p = 0.0015). A trend towards decreased anterograde velocities are also seen with APPswe but this is not significant (NS, p = 0.154). Box plots outline the distribution of duration-weighted segmental velocities for each genotype. The horizontal bar represents the median. The upper and lower box edges represents 75% percentile (i.e. upper quartile) and 25% percentile (i.e. lower quartile), respectively. Note that motility analysis was calculated from net anterogradely and retrogradely moving vesicles and reversing vesicles. **F:** Significant increases in pause frequencies in both anterograde (p = 0.0058) and retrograde (p = 0.0454) vesicles are seen in TKV-GFP;APPswe larvae compared to TKV-GFP larvae. **G:** Significant increases in anterograde pause durations were also observed in TKV-APPswe (p = 0.0179) larvae compared to TKV-GFP larvae. Table 4 shows the summary of *in vivo* measurements obtained from genotypes TKV-GFP and TKV-GFP;APPswe.

Strikingly, TKV movement was also disrupted in larvae expressing APPswe or pathogenic polyQ (Figure 8A–C, Movie S4, S5), similar to what was observed with 50% reduction of motors (Figure 8A). Expression of APPswe caused TKV-GFP to accumulate into large GFP blockages (Figure 8B, bottom kymograph) and reduced the motility of TKV-GFP (Figure 8B, top kymograph). Quantification analysis of TKV-GFP movement dynamics showed significant decreases in TKV motility with APPswe (Figure 8D–G). Cargo population analysis showed significant increases in the population of stalled or static TKV vesicles (p = 6.64×10^−11^) and decreases in anterogradely moving vesicles (p = 4.75×10^−6^) and reversing vesicles (p = 5.05×10^−6^, Figure 8D, Table 3) with APPswe. Decreases in both anterograde and retrograde velocities were also seen, although only the retrograde velocity changes were significant (Figure 8EG, p = 0.0015). The average retrograde duration weighted segmental velocity was decreased from 0.585 µicons/sec to 0.280 µicrons/sec (Figure 8H). These retrograde velocity decreases were due to increases in both pause duration and pause frequencies although only pause frequency changes were significant (Figure 8F–H, p = 0.0454). Although no significant changes in anterograde velocities were observed with APPswe, both the anterograde pause duration (Figure 8F, p = 0.0179, Table 4) and pause frequencies (Figure 8G, p = 0.0058, Table 4) were significantly increased. This is consistent with the proposal that increases in the frequency of pauses and the duration of pauses decreased the average run lengths without having a dramatic effect on anterograde velocities (data not shown). Taken together quantitative analysis of TKV-GFP motility indicates that APPswe disrupts the bi-directional movement of TKV-GFP. In contrast, expression of pathogenic polyQ severely perturbed the movement of TKV-GFP such that there were only very few slow moving TKV-GFP vesicles. While large GFP blockages were seen, the presence of TKV-GFP vesicles was greatly decreased in larval axons expressing pathogenic polyQ in contrast to larvae expressing TKV-GFP alone (Figure 8AC). However, although slow moving TKV-GFP complexes were observed (Figure 8C, top kymograph), we were unable to reliably quantify TKV-GFP dynamics in the context of pathogenic polyQ since our custom particle tracker program, which automatically tracks vesicle trajectories was unable to detect whole vesicle tracks (a criteria for our motility analysis [57]). Taken together, our observations indicate that while APPswe significantly disrupts the movement of BMP proteins, pathogenic polyQ severely perturbs the movement of BMP proteins. Thus the transport defects mediated by these two disease proteins disrupts BMP signaling and likely contributes to the synaptic morphological abnormalities observed in both APPswe and pathogenic polyQ NMJs (Figure 3).

**Table 3:** Summary of the number of vesicles for genotypes TKV-GFP and TKV-GFP;APPswe.

	TKV-GFP	TKV-GFP;APPswe
Total number of vesicles	184	181
Stationary vesicles	36	145
		p = 6.64×10^−11^ ***
Anterograde vesicles	83	21
		p = 4.75×10^−6^ ***
Retrograde vesicles	2	7
Reversing vesicles	63	8
		p = 5.05×10^−6^ ***

*Significance <0.05,

**significance <0.01,

***significance <0.001 as determined one way ANOVA followed by Bonferroni's test.

**Table 4:** Summary of *in vivo* measurements from the customized single particle tracking software program obtained from genotypes TKV-GFP and TKV-GFP;APPswe.

	TKV-GFP	TKV-GFP;APPswe
Anterograde duration weighted segmental velocity (mean±SEM; µm/sec.)	0.402±0.024	0.340±0.033
Retrograde duration-weighted segmental velocity (mean ±SEM; µm/sec.)	0.585±0.075	0.280±0.044
		p = 0.0015**
Anterograde segmental pause frequency (mean±SEM; pause/sec.)	0.039±0.004	0.060±0.006
		p = 0.0058**
Retrograde segmental pause frequency (mean ±SEM; pause/sec.)	0.039±0.004	0.060±0.006
		p = 0.0454*
Anterograde pause duration (mean ±SEM; sec.)	4.412±0.604	5.111±0.474
		p = 0.0179**
Retrograde pause duration (mean ± SEM; sec.)	3.68±0.713	5.575±0.993
		p = 0.291

Arrows indicate direction of change for TKV-GFP;APPswe compared to TKV-GFP.

*Significance <0.05,

**significance <0.01,

***significance <0.001 as determined by nonparametric Wilcoxon-Mann-Whitney test. Distributions were determined to be non-normal by Lilliefors test.

## Discussion

We have identified that perturbations in axonal transport disrupts the BMP signaling pathway, a pathway essential for synaptic formation, maintenance and function of the Drosophila NMJ. Our observations lead us to two main conclusions; 1) receptors and ligands of the BMP signaling pathway are transported within axons via an association with kinesin-1 and dynein motors, and 2) BMP signaling is disrupted in two neurodegenerative diseases as a consequence of axonal transport defects. These findings provide new insight into the pathological propagation of disease in two neurodegenerative diseases; that defects in long distant transport likely is the earliest contributor to the synaptic abnormalities and dysfunction observed in these two human neurodegenerative diseases.

### Axonal transport and BMP signaling

BMP signaling is a highly conserved pathway that is essential for organized assembly of synapses and is critical for coordinated growth of neurons during development in both invertebrates and vertebrates [70], [71]. In the Drosophila NMJ, BMP retrograde signaling is required for synaptic terminal growth and functional refinement. The muscle derived BMP ligand, Gbb, signals through neuronal receptors wit, tkv and sax [10], [38]–[41]. Receptor activation then leads to an increase in the phosphorylation of R-Smad, mad, at the NMJ terminals followed by nuclear translocation of p-Mad through its interaction with the co-Smad, med. Mutations of the members of this cascade show drastic reduction in the number of synaptic boutons and in the amount of neurotransmitter release at the NMJs [33], [39], [10], [40], including axonal transport defects [43], [69], [38]. However, although the neuronal derived BMP ligands and receptors are expressed in multiple cells in the CNS [10], [12], [13] and they function at the NMJs, the mechanism by which the retrograde signal is translocated into the nucleus has not yet been fully identified.

There are at least two possible mechanisms by which BMP signals move retrogradely. One possibility is that similar to the NGF-TrkA signaling endosome [11], components of the BMP pathway are trafficked within the axon in a signaling endosome (Figure S3). Studies have shown that BMP receptors colocalize with each other and with endosomal markers [67]. wit and tkv tagged GFP vesicles move bi-directionally within axons, however Mad tagged GFP appeared cytoplasmic [67] (Figure 7), and axonal blockages were observed with loss of function of tkv, mad, sax, and wit [69], [43], [38]. Yeast two hybrid analysis and binding assays identified that Tctex-1, the regulatory light chain of dynein binds BMPR-II, the mammalian wit orthologue [66]. Consistent with these results, our analysis showed that neuronal derived BMP receptors (wit, tkv, sax) and ligands (mad, med) functionally interact with both kinesin-1 and dynein motors (Figure 5, 6, 7) indicating that these components can be transported within the axon via an association with molecular motors (Figure S3). Normally p-Mad localization is observed in motor neuron cell bodies, axons and NMJs [72]–[73], [43], [39], [74]–[75] (Figure 4), and disruption of the dynein complex by either loss of roblk (Figure 4) or excess of DN Glued [10] perturbed BMP signaling, as measured by p-Mad. Further, a direct link between the activation of BMP signaling and the growth of presynaptic arbors has also been identified [76]–[77]. Ball et al demonstrated that Trio, the Rho-type guanyl-nucleotide exchange factor (GEF) is under the transcriptional control of BMP signaling and, together with Rac, is involved in presynaptic growth and regulation of neurotransmitter release [78]. In addition, loss of function of BMP components or motor proteins drastically reduced the number of synaptic boutons and the amount of neurotransmitter release [38], [39], [40], [10] (Figure 1). Therefore a BMP signaling endosome shares several key characteristics of the NGF-TrkA signaling endosome.

The second possibility is that while BMP receptors and ligands are anterogradely transported to the NMJs via kinesin (Figure 5, 6, 7), the retrograde signal might not be directly moved by a signaling endosome bound to dynein. Evidence for this proposal stems from a recent study that suggested that there could be at least two distinct molecular populations of p-Mad, the downstream signal for BMP retrograde signaling [67]; one at motor neuron cell bodies and one at NMJs. It was proposed that these distinct p-Mad populations are differentially phosphorylated by receptors at the synaptic terminal and cell body similar to the phosphorylation of the ERK isoforms [79]–[80], [67]. Smith et al [67] showed that while one p-Mad antibody identified both the cell body and NMJ p-Mad populations, two other p-Mad antibodies only recognized p-Mad at the cell bodies but not at the synapse. Perhaps p-Mad could bind different and distinct partners at the NMJs and motor neuron nucleus, and the nature of this binding could preclude access for some antibodies but not others. Alternatively, phosphorylation of Mad [81]–[82], [69] could activate p-Mad at nerve terminals, which would then activate downstream substrates, and it is these substrates that provide the retrograde signal to activate p-Mad at the cell bodies, not p-Mad itself. In this context, while loss of function of dynein would still eliminate the retrograde propogation of the activated signal, and disrupt activation of the cell body specific p-Mad population, the functional association we observed between Mad and dynein disagrees with this possibility. Thus the mechanisms of BMP signaling appear to be more complex than previously thought, and our work raises the possibility that perhaps most likely some combination of these events might exist. Therefore, further study would be needed to identify these mechanisms, the composition of the BMP vesicle complex and the putative p-Mad signaling endosome, as well as the potential downstream substrates of p-Mad.

### BMP signaling and Neurodegenerative disease

Defects in BMP signaling as a pathological mechanism has been proposed in several neurodegenerative diseases. BMP signaling has been reported to be decreased in Amyotrophic Lateral Sclerosis (ALS), Spinal muscular atrophy (SMA) and HD, while BMP signaling was increased in Hereditary Splastic Paraplagia (HSP) and Multiple Sclerosis (MS) [83]. As demonstrated by our study these BMP signaling defects could likely occur via perturbation in long distance transport since many of these diseases also show axonal transport defects [23], [43], [84]–[87] (Figure S3). Studies found that loss of survival motor neuron (smn) function, a gene that causes SMA, reduced the levels of p-Mad, suggesting that smn may play a role in BMP signaling [88]. Intriguingly, the same type of mutation (P58S) in VapB, a gene involved in familial ALS also affected BMP signaling. VapB overexpression resulted in increased levels of p-Mad, while loss of VapB decreased the levels of p-Mad compared to controls [89]. In addition, patients with HD accumulated high levels of CIP4, a huntingtin interacting protein [90]. CIP4 is thought to down regulate BMP signaling, since CIP4 mutants secreted excessive Gbb into the extracellular space. Thus the excess CIP4 found in HD brains could result from defects in transport and may lead to the down regulation of BMP signaling that we (Figure 4,5,8, S3) and others observe [83], contributing to the dystrophic striatal and corticostriatal neurites observed in HD [23].

A possible link between HSP proteins and BMP signaling was also revealed in a study using spichthyin (spict), the fly ortholog of the HSP gene, Nonimprinted in Prader-Willi/Angelman (NIPA1). In spict mutants the level of p-Mad was increased by 4-fold and mutations in tkv, sax, wit, gbb, and the co-Smad medea suppressed the spict synaptic bouton overgrowth phenotype [43]. Interestingly in mammals, NIPA1 appears to function as an inhibitor of BMP signaling. Although the mechanism of function is still unclear, knockdown of Spastin and Spartin, two other genes involved in HSP resulted in an almost identical increase in p-Mad levels as observed for NIPA1 mutants [91]–[92]. Mutations in Atlastin-1, another HSP gene, exhibited a dominant-negative effect on the trafficking of BMPRII (wit), disrupting BMP signaling [86]. Intriguingly, these HSP gene mutants also exhibited axonal transport defects [43], [86], [87], suggesting that perturbations in long distant transport likely is a common mechanism in HSP disease pathology. Increased levels of BMP6 have also been observed in AD brains and in transgenic AD mice together with impaired neurogenesis [93]. Therefore defects in long distant transport likely is the earliest contributor of the synaptic abnormalities and dysfunction seen in many neurodegenerative diseases. Thus our study highlights a potential novel therapeutic pathway for early treatment, prior to neuronal loss and the occurrence of clinical symptoms of disease.

## Materials and Methods

### Drosophila Genetics

Three loss of function Drosophila syt lines, amorphic syt^AD4^, syt^[N6]^ and hypermorphic syt^T77^ and the transgenic UAS-syt-EGFP line were used (Bloomington). These flies were raised as detailed in [62]. For loss of BMP signaling proteins, tkv^[7]^, wit[B11, sax^[5]^, med^[5]^, and mad^[1–2]^ mutant lines were used (Bloomington). For loss of motor protein function, khc^20^. klc^8ex94^, dhc64C^4–19^ and, robl^k^ mutant lines were used ([19], [20], [21]. Expression of human disease proteins human APPSWE and polyQ were done by crossing UAS-APPSWE (Gunawardena et al 2001) and UAS-MJD77Q lines to the pan neuronal GAL4 driver APPL-GAL4 at 29°C. For genetic interaction experiments, yw;T(2∶3) CyO TM6B, Tb/Pin^88K^ was used. The chromosome carrying T(2∶3) CyO TM6B, Tb is referred to as B3 and carries the dominant markers, Hu, Tb and CyO. For genetic interaction tests with kinesin and dynein motors, yw/yw;B3/Pin^88K^ females were crossed to khc^20^, klc^8ex94^, dhc64C^4–19^ or robl^k^ and motor protein mutant/T(2∶3) CyO TM6B,Tb males were crossed to BMP signaling mutants and females from this cross were used for analysis.

### Embryo and larval preparations, immunohistochemistry, and quantification

Embryo were collected, fixed and immunostained as described in [96]. Third instar larvae were dissected, fixed, and segmental nerve immunostainings were done as described [63], [94]. Briefly, larvae were dissected in dissection buffer (2× stock contains 128 mM NaCl, 4 mM MgCl_2_, 2 mM KCl, 5 mM HEPES, and 36 mM sucrose, pH 7.2). Dissected larvae were fixed in 4% formaldehyde and incubated overnight with antibodies against cysteine string protein (CSP, 1∶10, Developmental Studies Hybridoma Bank), DLG (1∶100, Developmental Studies Hybridoma Bank), p-Mad (1∶100, Dijke) and/or HRP-TR or HRP-FITC (1∶100 Invitrogen). Larvae were incubated in secondary antibodies (Alexa anti-mouse 568 or Alexa anti-rabbit 488, 1∶100, Invitrogen) and mounted using Vectashield mounting medium (Vector Labs). Images were collected using Leica TCS SP2 AOBS Spectral confocal microscope as described [63], [97]. Quantitative analysis on the extent of blockages and NMJ between muscle 6 and 7 at larval abdominal segments A4–5 was carried out by collecting six confocal optical images, from where several segmental nerves are visible or come into focus through the optical series. For axonal blockages for each genotype, ten animals were imaged, and at least four nerves were analyzed over a length of 50 µm, using the threshold, density slice, and particle analysis functions in NIH image software as previously described [63]. For NMJ analysis, ten animals were imaged and the type 1 NMJ between muscle 6 and 7 at larval abdominal segments A4–5 were imaged. The threshold, density slice, and particle analysis function in NIH image software was used to quantify parent and satellite bouton, and bouton areas. Synaptic length was measured in NIH image, within the corresponding muscle area to avoid any biases that might arise due to a variation in muscle size among different larvae and genotypes. Average numbers of parent and total boutons, and synaptic lengths and the % of satellite boutons were calculated and graphed using an EXEL worksheet. Using NIH image, p-Mad and HRP intensities were obtained and graphed using an EXEL worksheet.

### Statistics

For immunofluorescence analysis of axonal blockages and synaptic boutons statistical analysis was performed using 1) the two-sample two-sided student's T-test (Excel (Microsoft Corp) [63] and 2) ANOVA followed by post hoc analysis (SPSS Statistics 20 (IBM Corp.). Two other multiple comparison procedures (the Bonferroni and Dunnett procedures) specifically designed to compare each treatment to a control was also performed as previously done [63]. Differences were considered significant at a significance level of 0.05, which means a 95% statistically significant correlation. All three statistical methods revealed similar significant differences.

### *In vivo* microscopy of TKV vesicle movement

Drosophila transgenic lines expressing TKV-GFP was generated as previously described [57]. Females from this line were crossed to males from APPL-GAL4 or D42-GAL4, which express in all neurons. Female larvae were used for all *in vivo* imaging. To generate TKV-GFP with motor protein reduction, APPLGAL4;roblk or klc/B3 larvae, were crossed to males that were TKV-GFP and non tubby female larvae were used. To generate TKV-GFP with human APPswe or pathogenic polyQ77, APPL-GAL4:UAS-TKV-GFP/B3 larvae were generated and crossed to males that were UAS-APPswe or UAS-polyQ77 and non tubby female larvae were used. 3^rd^ instar larvae were dissected on a sylgard platform using Ca^2+^ free buffer containing the following, Nacl (128 mM), EGTA (1 mM), MgCl2 (4 mM), KCl (2 mM), HEPES (5 mM), and sucrose (36 mM) as described in Kuznicki et al [98]. Dissected animals were inverted onto a cover slip and imaged using a Nikon Eclipse TE 2000-U inverted microscope with a Coolsnap HQ camera and a 100×/1.40NA oil objective. 150 frames of videos were collected at 200 or 2000 ms exposure under the control of Metamorph software. For each genotype, four time-lapsed movies were collected for each animal; five animals were imaged; a total of 20 movies were collected and analyzed.

### *In vivo* movement analysis and statistics

*In vivo* movement analysis was performed as described in Gunawardena et al [57]. Briefly, GFP tagged vesicles/organelles from time lapsed videos of 150-frames imaged at 200 msec (50% kinesin-1 or dynein reduction and control) for a total of 30 sec per movie or 2000 msec (pathogenic polyQ and control) for a total of 30 sec per movie were quantified. Movies were taken at a 2×2 binning factor giving a spatial resolution of 0.126 micron/pixel. Four consecutive movies were imaged per cell. GFP vesicles were detected as single particles automatically and analyzed using a custom particle tracker program as detailed in [57]. For each genotype, individual cargoes were automatically classified as being either stationary, anterograde, retrograde, or reversing. Cargo trajectories of each genotype were then analyzed by calculating different descriptors that characterize the overall distribution of cargo population and individual cargo behavior in terms of velocity, pause, run lengths and reversals (switches). In particular, to determine velocity of a specific cargo, its trajectory is first partitioned into segments that are uninterrupted by pause or reversal events. For a given direction, either anterograde or retrograde, duration-weighted segmental velocity of the cargo is defined by its total distance of movement divided by its total duration of movement in that direction. This definition effectively weights cargo velocities within different segments by their durations. All data analysis was conducted using customized software written in MATLAB (Mathworks) and C++ and in depth details are provided in [57].

To evaluate statistical significance, as previously done, *in vivo* data were first checked for normality using three different tests implemented in the *nortest* package of *R*: the Lilliefors test, the Anderson-Darling test, and the Shapiro-Francis test [57]. For those that generally follow normal distributions, their means were compared using the two-sample two-sided student's T-test or ANOVA. For those following non-normal distributions, their means were compared using the permutation T-test or the Wilcoxon Mann-Whitney rank-sum test [57].

### Western blot analysis

As previously described 10 larval brains from each genotype (wit^[B11]^−/+, khc−/+, roblk−/−, and wild type were homogenized in acetate buffer (10 mM HEPES, pH 7.4, 100 mM K-acetate, 150 mM sucrose, 5 mM EGTA, 3 mM Mg-acetate, 1 mM DTT) with proteinase and phosphotase inhibitors [95]. Debris were removed by centrifugation at 1000× *g* for 7 min, and the resulting supernatant was analyzed using Western blotting. Antibodies wit monoclonal antibody (23C7, Developmental Studies Hybridoma Bank at 1∶500) and anti-tubulin monoclonal antibody (Abcam at 1∶1000 dilution) were used. Immunoreaction was detected using the ECL kit (Pharmacia) and imaged using QuantityOne (Bio-Rad). Quantification analysis was performed using NIH ImageJ software. Each lane of the gel was analyzed using plot lane, wand, and label peaks gel analysis functions. Data obtained as percent values for each sample by Image J were analyzed in Excel (Microsoft Corp.). Relative intensity was calculated by dividing the percent value for each sample by percent value of tubulin and then normalized to wild type, so that wild type was 1. Using the two-sample two-sided student's T-test or ANOVA, differences were considered significant at a significance level of 0.05, which means a 95% statistically significant correlation from three separate membranes from three different experiments.

## Supporting Information

Figure S1 **Expression of APPswe increases the number of satellite boutons.** Larvae expressing APPswe show increased numbers of satellite boutons (arrows) protruding from parent boutons (arrow heads). Bar = 10 µm
(TIF)

Figure S2 **Axonal blockages are observed in embryonic neurons from motor protein mutants.** Stage 15–17 embryos were fixed and stained with synaptic vesicle markers CSP. Axonal accumulations are observed as brightly stained punta (arrows) in embryonic neurons from roblk−/− embryos. In wild type embryos embryonic neurons are smoothly stained.
(TIF)

Figure S3 **Working model for the movement of BMP components within axons.** Our observations support a working model in which BMP components are transported within vesicles to the axonal terminal in a kinesin-dependent manner. BMP components (tkv, wit, sax, mad, med) are transported within a single vesicle or within different vesicles. A BMP signaling vesicle containing activated p-Mad is transported back to the cell body in a dynein-dependent manner, similar to the NGF-TrkA signaling vesicle. In disease states defects in transport (X) decreases kinesin-mediated transport of BMP components to the nerve terminal and reduces dynein-mediated transport of BMP signals to the cell body.
(TIF)

Movie S1 **Movement dynamics of TKV-GFP vesicles in normal larval nerves.** Frame rate: 0.2 sec/frame. Display rate: 30 frames/sec.
(MOV)

Movie S2 **Movement dynamics of TKV-GFP vesicles in larval nerves in the context of roblk−/+.** Note that TKV-GFP blockages are observed. Frame rate: 0.2 sec/frame. Display rate: 30 frames/sec.
(MOV)

Movie S3 **Movement dynamics of TKV-GFP vesicles in larval nerves in the context of klc−/+.** Note that TKV-GFP blockages are observed. Frame rate: 0.2 sec/frame. Display rate: 30 frames/sec.
(MOV)

Movie S4 **Movement dynamics of TKV-GFP vesicles in larval nerves in the context of APPswe.** Note that TKV-GFP blockages are observed. Frame rate 2 sec/frame. Display rate 30 frames/sec.
(MOV)

Movie S5 **Movement dynamics of TKV-GFP vesicles in larval nerves in the context of polyQ77.** Note that TKV-GFP blockages are observed. Frame rate 2 sec/frame. Display rate 30 frames/sec.
(MOV)
