# Supplementary figures and images for: Disruption of Axonal Transport Perturbs Bone Morphogenetic Protein (BMP) - Signaling and Contributes to Synaptic Abnormalities in Two Neurodegenerative Diseases
Source: PLoS One. 2014 Aug 15;9(8):e104617. doi: 10.1371/journal.pone.0104617 (PMC4134223; doi:10.1371/journal.pone.0104617)

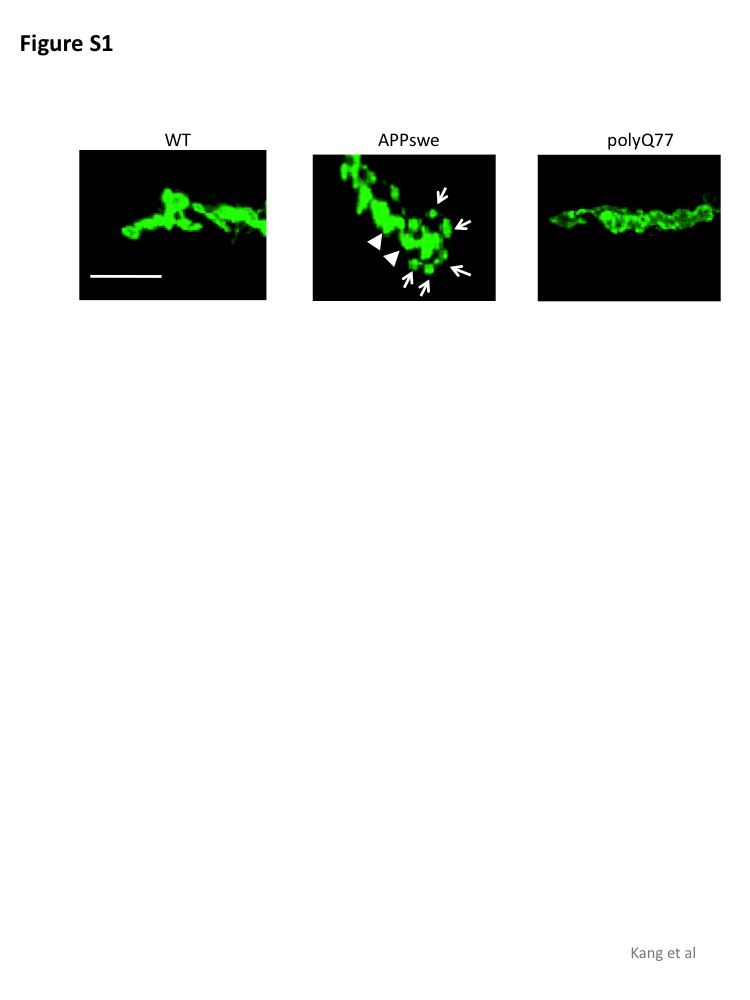

Supplement: Figure S1 — Expression of APPswe increases the number of satellite boutons. Larvae expressing APPswe show increased numbers of satellite boutons (arrows) protruding from parent boutons (arrow heads). Bar = 10 µm (TIF) [file pone.0104617.s001.tif]

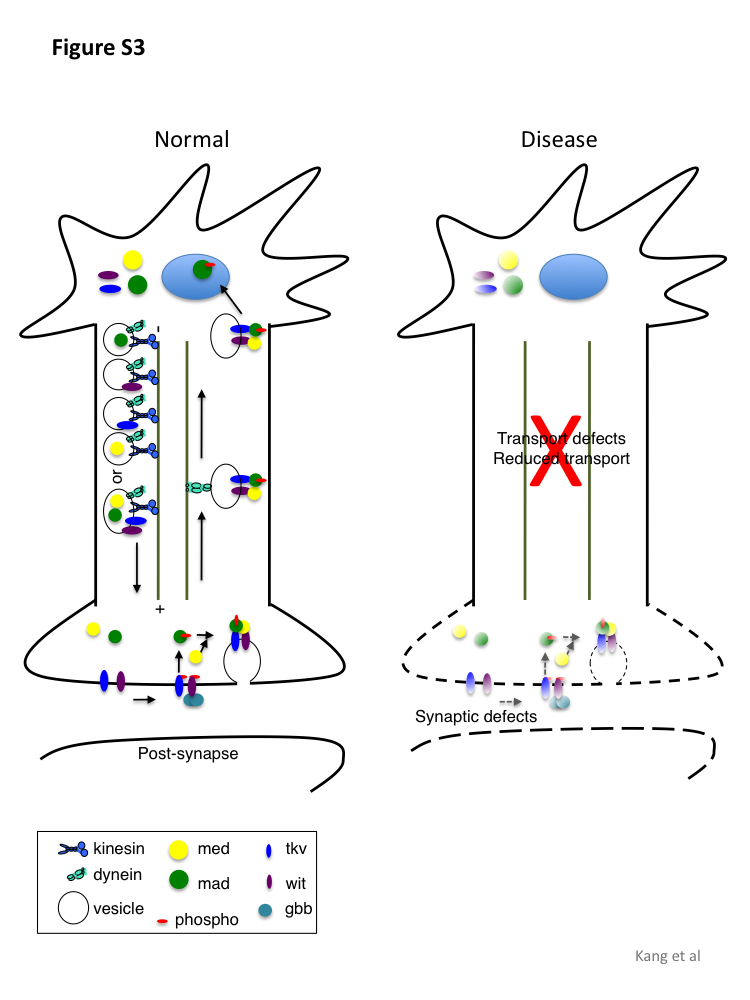

Supplement: Figure S3 — Working model for the movement of BMP components within axons. Our observations support a working model in which BMP components are transported within vesicles to the axonal terminal in a kinesin-dependent manner. BMP components (tkv, wit, sax, mad, med) are transported within a single vesicle or within different vesicles. A BMP signaling vesicle containing activated p-Mad is transported back to the cell body in a dynein-dependent manner, similar to the NGF-TrkA signaling vesicle. In disease states defects in transport (X) decreases kinesin-mediated transport of BMP components to the nerve terminal and reduces dynein-mediated transport of BMP signals to the cell body. (TIF) [file pone.0104617.s003.tif]
